# Supplementary material for: Population Pharmacokinetics of Isavuconazole in Critical Care Patients with COVID-19-Associated Pulmonary Aspergillosis and Monte Carlo Simulations of High Off-Label Doses
Source: J Fungi (Basel). 2023 Feb 6;9(2):211. doi: 10.3390/jof9020211 (PMC9960864; doi:10.3390/jof9020211)
Supplement: Supplementary file 1 [file jof-09-00211-s001.zip › jof-2139463-supplementary.pdf]

Supplementary Materials

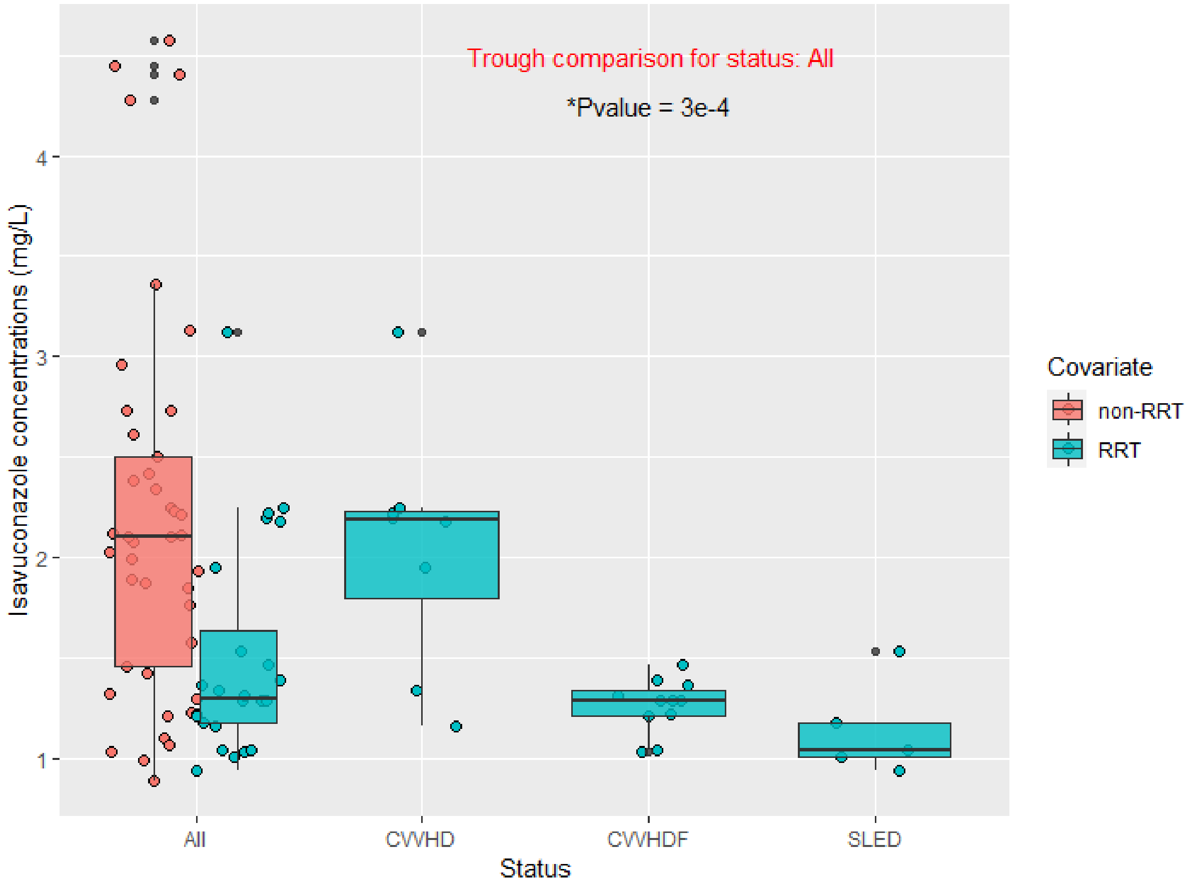

Figure S1. Variability comparison of trough concentrations between RRT and non-RRT.
**Welch two sample t-test*

Calculation S1. Wald test p-value calculation for covariate tagged as a regressor.

(1) To test if the shape of βCl is significantly different from 0:

$$H_0 : \beta\text{Cl} = 0 \text{ versus } H_1 : \beta\text{Cl} \neq 0$$

(2) The parameter estimate and the standard error (s.e) was used to calculate the Wald statistic (W):

$$W = \frac{\beta\text{Cl\_estimated - }\beta\text{Cl\_ref}}{s.e\_estimated} = \frac{0.45 - 0}{0.1} = 4.5$$

(3) W has to be compared to a standard normal distribution:
Rscript, pvalue = 2 * pnorm(W, mean = 0, sd = 1, lower.tail = FALSE) = 6.8e-6

Adapted from: https://monolix.lixoft.com/faq/complex-parameter-covariate-relationships-time-dependent-covariates/

Table S1. RRT parameters of patients receiving intravenous isavuconazole.

Patient	Number of days before treatment initiation	Number of days before sampling	CRRT/SLED machine	CRRT/SLED mode	Filter type	Blood flow rate (mL/min)	Effluent flow rate (L/h)
1	3	8	Fresenius 5008	SLED	Nephral ST400	200	0.2
2	-2	5	Fresenius Multifiltrate	CVVHD CiCa	Ultraflux AV 1000S	100	2
3	1	6	Fresenius Multifiltrate	CVVHD CiCa	Ultraflux AV 1000S	100	2
4	-5	-1	Fresenius 5008	SLED	Nephral ST400	200	0.2
5	9	19	Prismaflex	CVVHDF	AN69	200	3
6	-3	7	Fresenius multifiltrate	CVVHD CiCa	Ultraflux AV 1000S	150	3
